# Extraosseous Ewing Sarcoma Presenting with Inferior ST-Elevation Myocardial Infarction and Systemic Emboli due to Tumor Thrombus and Invasion of the Left Atrium

**DOI:** 10.1155/2020/3861927

**Published:** 2020-01-27

**Authors:** Jennifer Dotson, Madhulika Urella, Mina Shenouda, Ahmad Abu-Hashyeh, Yehuda Lebowicz

**Affiliations:** ^1^Marshall University Department of Hematology and Oncology, 1400 Hal Greer Blvd Huntington, WV 25701, USA; ^2^Marshall University Department of Internal Medicine, 1249 15th St, Suite 2000 Huntington, WV 25701, USA

## Abstract

Extraosseous Ewing sarcoma is an uncommon entity in the adult population. Cardiac metastases or local invasion of a tumor into the heart is a known but also infrequent occurrence for most malignancies. We present a case of a patient with a history of extraosseous Ewing sarcoma who presented to the emergency room with chest pain and was found to have an inferior ST-elevation myocardial infarction and systemic emboli and was found to have recurrence of sarcoma invading the left atrium.

## 1. Introduction

Ewing sarcoma is an uncommon malignancy in adults that most often presents as an undifferentiated primary bone tumor; less commonly, it arises in soft tissue (extraosseous Ewing sarcoma (EOEWS)). Both are part of a spectrum of neoplastic diseases known as the Ewing sarcoma family of tumors (EFT). Metastases at diagnosis are infrequent, occurring in less than twenty-five percent of patients. However, due to relapse rates with local therapy and tendency to metastasize, EFT is considered to be a systemic disease and usually requires multimodality therapy [1]. We present a patient with a history of previously treated EES five years prior, who presented to the emergency room with chest pain and dyspnea and was found to have an inferior ST-elevation myocardial infarction (STEMI) and evidence of systemic emboli occurring in the setting of recurrent Ewing sarcoma of the lung invading the left atrium.

## 2. Case Presentation

**Figure 1:**
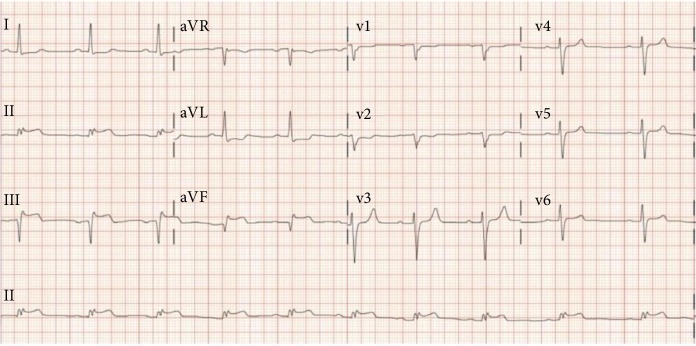
Electrocardiogram revealing ST-elevations in leads II, III, and AVF.

**Figure 2:**
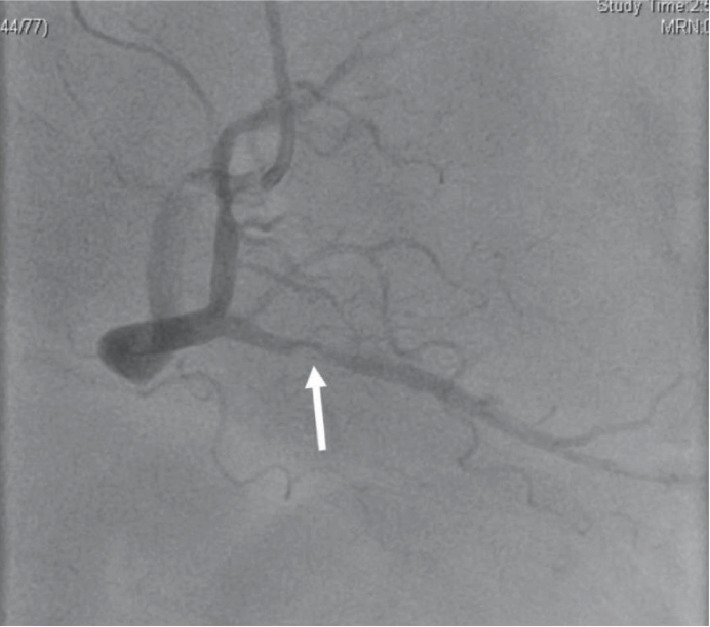
Left heart catheterization revealing marked stenosis of the RCA-PDA.

**Figure 3:**
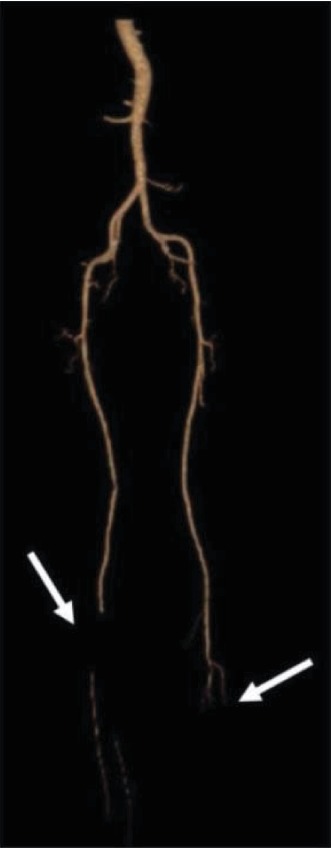
Computed tomography angiogram of the lower extremities showing a perfusion defect in the right tibial artery and left popliteal artery.

**Figure 4:**
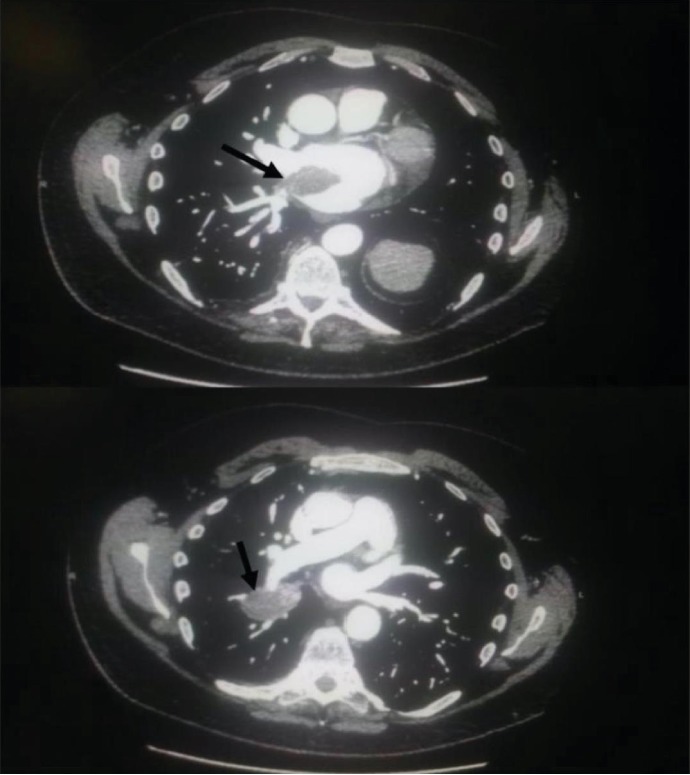
Computed tomography imaging showing a tumor in the lung parenchyma with invasion of the left atrium (black arrows).

**Figure 5:**
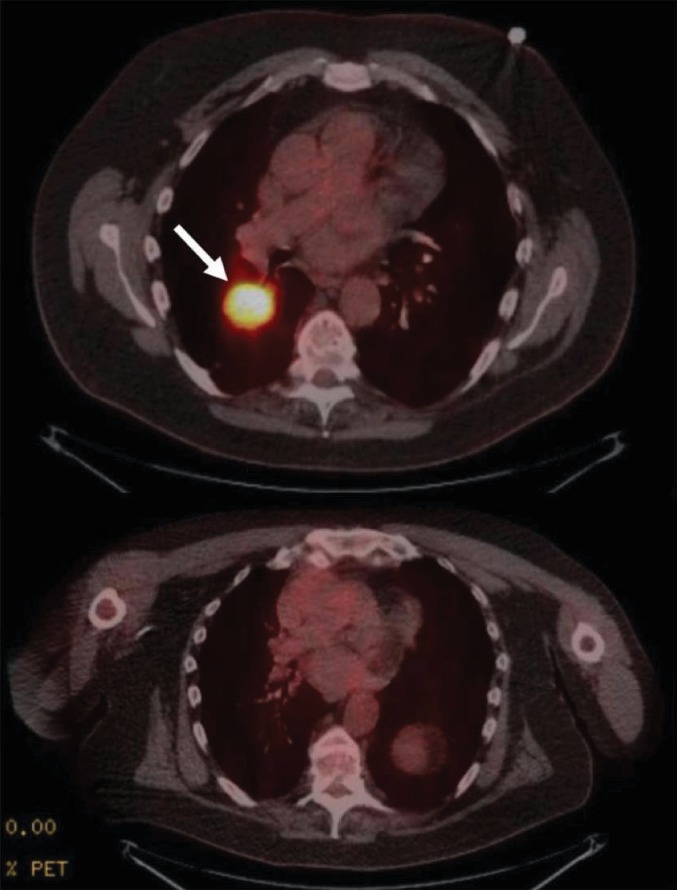
The top image is a positron emission tomography (PET) scan showing hypermetabolic mass abutting the left atrium. The bottom image shows resolution of hypermetabolic mass after six cycles of chemotherapy.

A 62-year-old male with past medical history significant for extraosseous Ewing sarcoma presented with chest pain and dyspnea to the emergency room. His electrocardiogram revealed inferior ST-elevation myocardial infarction (STEMI) (Figure 1). He was treated five years prior for an extraosseous Ewing sarcoma (EOEWS). At that time, he had presented with a rapidly growing left posterior neck mass, and biopsy revealed an infiltrative high-grade malignant neoplasm composed predominantly of small round blue cells. Immunohistochemistry was positive for cytokeratin AE1/AE3 and negative for leukocyte common antigen, CD56, chromogranin A, synaptophysin, thyroid transcription factor (TTF-1), cytokeratins 7 and 20, and cytokeratin 5/6. The cells were diffusely and strongly positive for CD99. Fluorescence in situ hybridization (FISH) showed a rearrangement of the EWSR1 gene in 58% of nuclei. Next-generation sequencing revealed a EWSR1-FLI1 fusion, along with a CHEK2 mutation and microsatellite stability. He had received systemic chemotherapy with vincristine, dactinomycin, and cyclophosphamide alternating with ifosfamide and etoposide, though he was unable to complete the full course of treatment due to side effects. This was followed by local therapy with radiation to the neck mass. He had a complete response and had been in remission from his disease since that time. At his current presentation with an acute STEMI, the patient underwent immediate left heart catheterization with percutaneous coronary intervention and drug-eluting stent placement to the obstructed right coronary artery/posterior interventricular artery (RCA-PDA, Figure 2). Echocardiogram revealed an ejection fraction of 45-50% with severe hypokinesis in the entire inferior, basal, middle, and inferior lateral apical septum and apical wall. After the procedure, the patient had also complained of claudication in his lower extremities. A CT angiogram was performed and revealed a perfusion defect in the right tibial artery and left popliteal artery as well as an incidental finding of a 4.6-centimeter right lower lobe lung mass. A computed tomography (CT) scan of the chest with intravenous contrast revealed a right perihilar mass with invasion of the left atrium and around four to five centimeters of tumor and/or bland thrombus within the left atrium (Figure 3). Additional CT imaging of the abdomen revealed splenic infarcts. The patient was started on intravenous heparin for anticoagulation in addition to clopidogrel for the drug-eluting stent. He underwent biopsy along with interruption of anticoagulation and antiplatelet therapy with bridging by heparin. A fine needle aspiration of the right lower lobe mass and bronchoalveolar lavage revealed insufficient material. He was discharged from the hospital on rivaroxaban and clopidogrel as it was deemed high risk for repeat biopsy and to withhold anticoagulation and antiplatelet therapy. An outpatient positron emission tomography (PET) scan revealed neoplastic uptake within the right lower lobe lung mass measuring five centimeters along with a standardized uptake value (SUV) of 13.4 with no other sites of metastatic disease (Figure 4). Almost a month after STEMI/stent placement, subsequent CT angiogram revealed a five-centimeter right lower lobe lung mass invading the left atrium. There was tumor versus bland thrombus in the left atrium measuring at 5 × 3.4 × 3.7 centimeters. As an outpatient, the patient continued to have ongoing claudication, and we presume this was from showering of emboli from thrombus. Due to the urgent need for initiation of chemotherapy and logistics of obtaining another biopsy with bridging of intravenous anticoagulation and antiplatelet therapy, we decided to start treatment with presumption of recurrent Ewing sarcoma, especially in light of proximity of the new mass to his neck mass four years prior. The patient was then started on chemotherapy with a regimen of vincristine, doxorubicin, and cyclophosphamide alternating with ifosfamide and etoposide with a near complete response after six cycles of therapy (Figure 5).

## 3. Discussion

Extraosseous Ewing sarcoma (EOEWS) with invasion of the left atrium occurring simultaneously with systemic emboli is a highly unusual presentation of this disease. EOEWS compromises only fifteen percent of bone sarcomas. The median age of diagnosis is typically around ten to fifteen years old, and less than five percent of cases occur in patients over the age of forty [1]. EOEWS is part of the Ewing sarcoma family of tumors (EFT). Recent data suggest that patients with EOEWS have slightly superior outcomes compared with patients with osseous Ewing sarcoma [2]. The incidence of EES is higher in older patients, especially females. Standard management of extraosseous Ewing sarcoma involves chemotherapy coupled with local therapy in the form of surgery and/or radiation therapy. Local therapy alone leads to relapse rates of 80-90%, but fortunately, Ewing sarcoma is typically highly responsive to chemotherapy and radiation therapy [3]. Though fewer than 30% of patients have metastases at the time of diagnosis, EFT is always considered a systemic disease due to its tendency for early dissemination. Ewing sarcoma spreads primarily to the lungs, with extrapulmonary metastases to the bone, liver, and brain being less common [1, 2].

Cardiac metastases in this disease are exceedingly rare and may be either primary or secondary, either from metastases or local invasion. The incidence of primary cardiac tumors is around 0.02%, while secondary tumors are more common, occurring one hundred times more frequently [4–6]. Most secondary tumors arise from primary malignancies such as lung and breast cancer. The most common secondary or metastatic cardiac tumor is melanoma in over half of reported cases, followed by breast, lung, and esophageal cancer, as well as lymphomas and leukemia [3, 5]. While rare, there are a few reports of cardiac metastases in adult Ewing sarcoma with involvement of the right ventricle and the pericardium, with two cases each [7, 8]. There are no reported cases in the literature involving the left atrium. There were instances in the literature of left-sided metastases causing systemic emboli in other types of cancers, however. Left-sided cardiac tumors may result in compression of coronary arteries or tumor thrombus formation whereas right-sided cardiac metastases may cause pulmonary embolism [5]. Cardiac tumors or metastases may cause heart failure, valvular disease, or conduction abnormalities. Tumor or bland thrombus may also cause embolism leading to syncope or death. Thus, myocardial ischemia may occur secondary to either extrinsic compression or from an embolus in the coronary arteries, the latter being the likely cause in our case. Our patient presented with simultaneous occurrence of acute myocardial ischemia in addition to peripheral extremity emboli on CT angiogram and evidence for splenic infarction. PET/CT imaging revealed a PET-avid mass in the lung parenchyma as well as hypermetabolic activity in the left atrium. PET avidity of the mass and left atrium is suggestive of left atrial invasion as well as likely component of thrombus either from the tumor itself or from the external compression of the atrium causing stasis of the blood. It is difficult to prove logistically if the myocardial infarction and peripheral emboli came from the tumor or actual blood thrombus without pathologic confirmation from a vessel biopsy. Due to this, he was anticoagulated and began on treatment. Additionally, while we were not able to confirm definitive biopsy of the recurrence, the left atrial mass had an excellent clinical response to chemotherapy that is typically given in Ewing sarcoma tumors. Although biopsy of recurrent presumed EOEWS invading the heart was not obtained, the response to chemotherapy suggests the same tumor. This would be in contradistinction to treatment for a new primary lung malignancy, which would be on the differential, as this is usually treated with a platinum chemotherapy doublet therapy. We believe that any patient with a history of cancer who presents with evidence of systemic emboli must be considered for possible cardiac metastases and possibility of subsequent tumor or bland thrombus.
